# Three global conditions for biodiversity conservation and sustainable use: an implementation framework

**DOI:** 10.1093/nsr/nwz136

**Published:** 2019-09-12

**Authors:** Harvey Locke, Erle C Ellis, Oscar Venter, Richard Schuster, Keping Ma, Xiaoli Shen, Stephen Woodley, Naomi Kingston, Nina Bhola, Bernardo B N Strassburg, Axel Paulsch, Brooke Williams, James E M Watson

**Affiliations:** 1 Beyond the Aichi Targets Task Force IUCN World Commission on Protected Areas and Yellowstone to Yukon Conservation Initiative, Canada; 2 Department of Geography and Environmental Systems, University of Maryland, USA; 3 Natural Resources and Environmental Studies Institute, University of Northern British Columbia, Canada; 4 Department of Biology, Carleton University, Canada; 5 State Key Laboratory of Vegetation and Environmental Change, Institute of Botany, Chinese Academy of Sciences, China; 6 IUCN World Commission on Protected Areas, Canada; 7 UN Environment Programme World Conservation Monitoring Centre (UNEP-WCMC), UK; 8 Conservation and Sustainability Science Centre, Department of Geography and the Environment, Pontifícia Universidade Católica and International Institute for Sustainability, Brazil; 9 Institute for Biodiversity, Germany; 10 School of Earth and Environmental Sciences, University of Queensland, Australia; 11 Wildlife Conservation Society, USA; 12 Centre for Biodiversity and Conservation Science, University of Queensland, Australia

‘Nature and its vital contributions to people, which together embody biodiversity and ecosystem functions and services, are deteriorating worldwide’ [1].

The United Nations Convention on Biological Diversity (CBD) is intended to ensure conservation of biodiversity, its wise use, and sharing of benefits from use of genetic resources. Through it, the Strategic Plan (SP) for Biodiversity 2011–2020 was created to make progress toward a vision of humanity ‘Living in Harmony with Nature’ by 2050 [2]. When that vision is realized, biodiversity will be valued, conserved, restored, and wisely used, so it can maintain ecosystem services and sustain a healthy planet, delivering benefits essential for all humans (2050 Vision). The SP contains 20 global targets (the Aichi Targets) and applies to other nature-oriented UN Conventions.

Related to this, in 2015, the UN created the Sustainable Development Goals (SDGs), an overarching plan for people, planet, and prosperity designed to achieve a multi-faceted vision, which includes living in harmony with nature. The SDGs stressed international cooperation, referenced biodiversity and climate throughout, and reaffirmed Rio Principle 7, which states that countries have common but differentiated responsibilities for the health of the ‘earth ecosystem.’ [3].

Confronted with the global crisis facing nature, the Parties to the CBD will meet in Kunming, China in October 2020. They have called for assistance in developing realistic baselines and frameworks that will support ambitious and measurable targets for a Post-2020 SP relevant to the SDGs that will make progress toward the 2050 Vision [4]. We offer this response.

Three Global Conditions for Biodiversity Conservation and Sustainable Use (3Cs) is an implementation framework suitable for use in the Post-2020 SP. It follows the well-known drivers-state-pressure-response approach for addressing biodiversity conservation on land [5]. A compatible marine approach is under development.

The 3Cs framework evaluates land-use drivers and human pressures to establish a baseline state of three broad terrestrial conditions: Cities and Farms cover 18% of land (C1), shared lands 56% (C2), and large wild areas 26% (C3). It maps all but Antarctica (Fig. 1) and enables development of suites of conservation responses and production practices appropriate for each condition that are clustered on a continuum from those appropriate to the most heavily impacted areas to those best suited to the wildest areas remaining on Earth. These include:

**C1:** Increase conservation efforts to secure endangered species and protect all remaining primary ecosystem fragments. Mainstream sustainable practices such as protecting good farmland, practicing productive regenerative agriculture, and keeping nitrogen out of freshwater. Maintain pollinators and increase ecological restoration. ‘Green’ cities to reduce carbon emissions, prevent urban sprawl, and provide access to nature for urban dwellers’ health and well-being.

**C2:** Establish ‘ecologically representative and well-connected systems of protected areas (PAs)’ while increasing coverage of key biodiversity areas (KBAs); restore and maintain ecological processes and viable populations of native species (ensure area protected is in the range of 25%–75% per ecoregion) [6]. Across landscapes integrate sustainable natural resource extraction and activities such as tourism, grazing, and use of wildlife (where appropriate and sustainable) with indigenous knowledge and well-managed, equitable, and properly funded PA networks.

**Figure 1.**
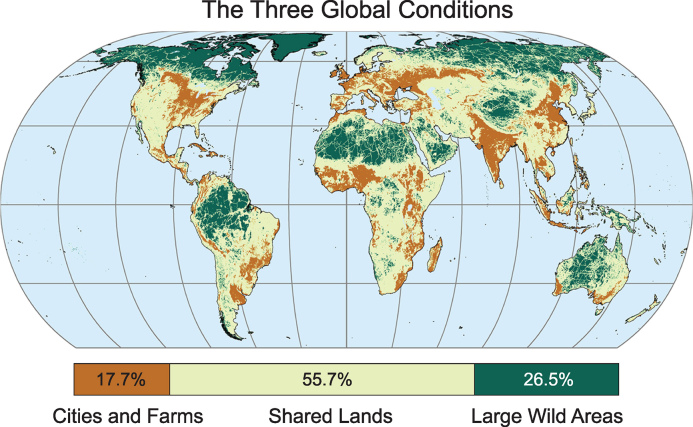
Map of the Three Global Conditions, with their relative global areas illustrated in bar at bottom (Supplemental Methods). Eckert IV projection.

**Table 1.** The three global conditions, summary statistics (computations and sources in Supplementary Methods).

	Cities and farms	Shared landscapes	Large wild areas	Whole world
Distribution of land	17.7%	55.7%	26.5%	100.0%
Distribution of human population (2015)	75.2%	24.7%	0.1%	100.0%
Percent of area protected	5.8%	14.4%	24.4%	15.5%
Distribution of key biodiversity areas	10.5%	64.9%	24.6%	100.0%
Food calories produced by farming and ranching	72.0%	27.8%	0.3%	100.0%
Percent global area under indigenous management or tenure	7.8%	48.6%	43.6%	100.0%
Average number of vertebrate species per 100 km^2^ area	228.9	193.3	102.3	175.0
Average number of threatened vertebrate species per 100 km^2^ area	6.9	5.6	3.3	5.2
Median forest aboveground biomass carbon density, tonnes/ha	13.2	40.1	36.8	33.5
Median soil organic carbon density, tonnes/ha	45.8	42.7	53.0	45.8

**C3:** Retain overall ecological integrity and associated global processes such as carbon storage and rainfall generation, fluvial flows, and large migrations; prevent further fragmentation allowing only rare nodes of intense industrial development enveloped in a largely wild matrix. Remove and restore anomalies. Establish large PAs and indigenous and community conserved areas. Secure indigenous knowledge and livelihoods.

Most of these responses and practices are already found in the current Aichi Targets (see Supplementary Text in Supplementary Materials). Some actions identified for one condition may be applicable in another. In addition, ecological connectivity should be secured across all three conditions for resident and migratory species and for resilience to climate change.

Intended for simultaneous use, these conservation responses and sustainable practices form a coherent basis for common national actions and international cooperation for ambitious efforts to protect the ‘earth ecosystem.’ Countries with similar conditions have similar responsibilities and options for domestic action. Developed nations can also support efforts elsewhere, especially when their trade footprints cause biodiversity loss in other countries.

To make the map (Fig. 1), we combined global maps of intensive human land uses [7] and eight human-caused pressures on land determined by the Human Footprint v 2.0 [8].

A break point of half the land transformed by cities, cultivation, and intensive grazing was set for the boundary between C1 and C2. For the boundary between C2, which has both significant untransformed natural conditions and substantial land uses, and C3, which is still predominantly natural with light land use, we used a human footprint pressure of <4 out of 50 and intensive land use covering <0.5% of regional landscape hexagons of ∼100 sq. km. (details in Supplementary Methods in Supplementary Materials). Within each of the conditions might be found small elements of other conditions.

Conservation and human-use values vary by condition. The relative distribution of human population, above- and below-ground carbon, existing PAs, distribution of vertebrates and threatened vertebrates, KBAs, indigenous interests in land, and food calories produced are summarized by condition in Table 1 (source data and analysis methods described in Supplementary Materials). The concentration of people, food production, and threatened vertebrates in highly productive C1, the abundance of KBAs and PAs that could be interconnected compatibly with natural resource extraction in C2, and the prevalence of carbon-rich soils and forests, small human populations, and indigenous management in C3 exemplify why it is useful to sort conservation strategies by varied societal and natural conditions.

We acknowledge that these boundaries are fuzzy and that national maps should be improved through calibration and expert ground-truthing. Nevertheless, these generalizations hold true enough to be useful for developing regional and global-scale strategies.

The Post-2020 SP will be negotiated politically, not by scientists. The 3Cs are scientifically grounded yet easy for a non-scientist to visualize and understand. Maps of the 3Cs can be viewed by decision-makers on their hand-held devices where they can compare their conditions with those of other countries (global, regional, and national maps are at naturebeyond2020.com). The maps can be updated and monitored over time.

The 3Cs provide a coherent framework for countries to commit to global goals through realistic measures suitable for their current national conditions. It provides a basis for common but differentiated responsibilities for international cooperation to protect the earth ecosystem that can also serve as a guide for the participation of non-state actors. If implemented simultaneously, the strategies and actions identified by the 3Cs would be transformational steps toward securing biodiversity and realizing the 2050 Vision.

## Supplementary Material

nwz136_Supplemental_File
