# Supplementary material for: Three global conditions for biodiversity conservation and sustainable use: an implementation framework
Source: Natl Sci Rev. 2019 Sep 12;6(6):1080–2. doi: 10.1093/nsr/nwz136 (PMC8291457; doi:10.1093/nsr/nwz136)
Supplement: nwz136_Supplemental_File [file nwz136_supplemental_file.pdf]

Supplementary Materials: The Three Global Conditions

Please Cite: Locke et. al. 2019. Three Global Conditions for Biodiversity Conservation and Sustainable Use: an area-based framework for use in the Post-2020 Strategic Plan for Biodiversity. *National Science Review*. (DOI:10.1093/nsr/nwz136)

This document includes:

- **Supplementary Text 1: Fourteen Aichi Targets Enabled**
- **Supplementary Methods: The Three Global Conditions**

Supplementary Text 1: Fourteen Aichi Targets Enabled

Three Global Conditions conservation strategies provide a framework for:

- National planning (T2, T17), area-based conservation (T10, T11), and avoidance of fragmentation and degradation (T5), restoration (T14, T15), carbon storage and climate adaptation (T15), and extinction prevention (T12).
- Sustainable production (T4, T7), pollution reduction (T8), and mainstreaming (T1) and mobilizing financial resources (T20).
- Indigenous and local communities (T18)

Supplementary Methods: The Three Global Conditions
This document provides full details of the methods used to map the three global conditions and for subsequent analysis of ancillary variables across the three global conditions.

Produced by Erle C. Ellis (ece@umbc.edu), June 13, 2019

Please Cite: Locke et. al. 2019. Three Global Conditions for Biodiversity Conservation and Sustainable Use: an area-based framework for use in the Post-2020 Strategic Plan for Biodiversity. *National Science Review*. (DOI:10.1093/nsr/nwz136)

Contents of this Document

I. General Methods Description
- I.1. Three Global Conditions: Classification Rubric
- I.2. General Methods

II. Mapping the Three Global Conditions
- II. 1. Mapping Data Inputs
- II. 2. Detailed Three Conditions mapping methods, using ArcGIS 10.6

III. Analysis of ancillary variables across the Three Global Conditions
- III.1. List of ancillary variables analyzed
- III. 2. Computation of ancillary variables in DGG cells, using ArcGIS 10.6
- III. 3. Computation of ancillary variables across the three global conditions

IV. References Cited

I. General Methods Description

I.1. Three Global Conditions: Classification Rubric
1: Cities and Farms: Intensive land use > 50%
2: Shared Landscapes: Human Footprint >= 4 AND (intensive land use <= 50% AND >= 0.5%)
3: Large Wild Areas: Human Footprint Index < 4 AND intensive land use < 0.5%

I.2. General Methods
Regional landscapes across all continents except Antarctica were classified into three classes, the "three global conditions", using the rubric above, based on data for land use for year 2015 from HYDE 3.2.1 (Klein Goldewijk et al. 2017) and 2013 data for the Human Footprint (HFP;(Venter et al. 2016). Regional landscapes were mapped based on a set of 1,434,246 equal area hexagons of approximately 100 km^2 (96.19 km^2) based on a discrete global grid system ("DGG cells"; Level 12 ISEA Aperture 3 Projection (Sahr et al. 2003, Schmill et al. 2014). Percent land use areas, HFP, and other variables were computed for each DGG cell using zonal statistics in ArcGIS 10.6. Percent "intensive land use" area in each DGG cell was computed as the sum total of percent urban area, percent cultivated area, and percent intensively managed pasture area based on HYDE 3.2.1. HFP was computed as the maximum value within each DGG cell. DGG cells were then allocated to each of the three conditions classes using the Rubric above. Following the classification of the three global conditions, a set of ancillary variables were computed for each DGG cell using Zonal statistics in ArcGIS, as described in more detail below.

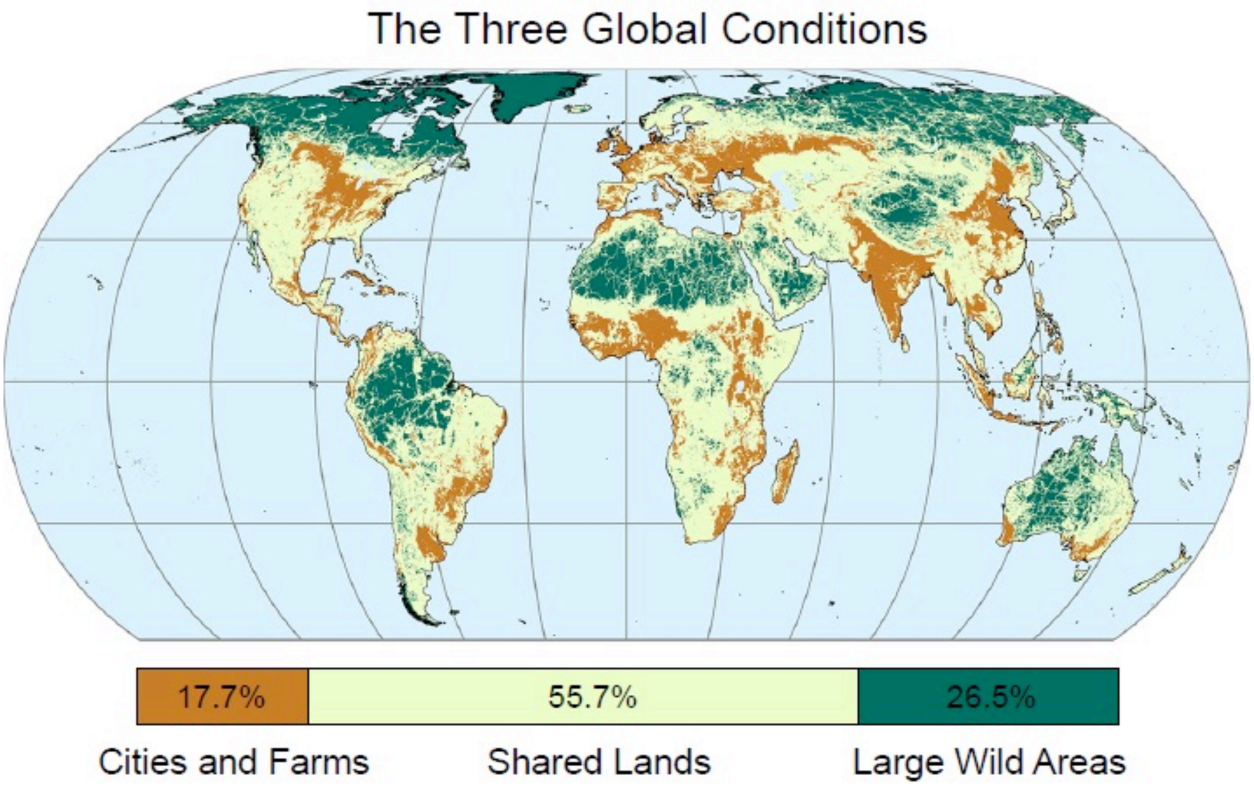

II. Mapping the Three Global Conditions

II. 1. Mapping Data Inputs

- HYDE 3.2.1 land use data for year 2015 as 5 arc minute geographic ASCII GRID format (5M grid cells) (Klein Goldewijk et al. 2017).
- Human Footprint (HFP) for year 2013 as 1 km^2 resolution raster in Mollweide projection GeoTiff format (Venter et al. 2016).
- Regional landscapes as hexagonal grid cells in shapefile format = L12 Discrete Global Grid cells ("DGG"; 96.19 km^2 hexagons (Sahr et al. 2003, Schmill et al. 2014)). Includes 1,434,246 DGG cells total. This is "**DGG shapefile**" in analysis below.

II. 2. Detailed Three Conditions mapping methods, using ArcGIS 10.6

1. Acquire and process HYDE 3.2.1 dataset (Klein Goldewijk et al. 2017).
    a. Compute % of each 5M cell covered by urban, crops, pasture by dividing land use areas by total land areas of each cell (divide by land area in each 5M cell = maxln_cr.asc layer).
    b. p_past = % intensive pasture use.
    c. p_cult = % crop area.
    d. p_urbn = % urban area.
2. Compute % intensively used area within each DGG cell.
    a. Compute P_USED raster = p_past + p_cult + p_urbn
    b. Compute P_USED within each DGG cell using zonal statistics, after converting DGG shapefile into 30 arc second GRID aligned with HYDE GRIDs.
    c. Add Mean statistic for P_USED to DGG shapefile.
3. Acquire Human Footprint (HFP) for year 2013 (Venter et al. 2016).
4. Compute HFP within each DGG cell.
    a. Compute statistics for HFP within each DGG cell using zonal statistics, after converting DGG shapefile into 1 km2 GRID aligned with HFP GRID.
    b. Add Maximum statistic for HFP, = "HFP_MAX" to DGG shapefile.
5. Compute three conditions variable ("3_cond_v4" field in DGG shapefile), using Rubric, above, in Field Calculator.
    a. threecond = 2
    b. IF [P_USED] > 0.5 THEN
    c. threecond = 1
    d. End IF
    e. IF [HFP_MAX] < 4 AND [P_USED] < 0.005 THEN
    f. threecond = 3
    g. End IF
6. Remove all DGG cells where data for HYDE or HFP was absent.
    a. Total of 1,434,246 classified DGG cells in final shapefile.
7. Produce map output as shapefile.
    a. Filename = **three_conditions_v4_map_only.shp**.
    b. Three conditions variable = "**3_cond_v4"** field.
    c. Compress in zip file: three_conditions_map_2019_06_12.zip
    d. This zip file represents the **Supplemental Data** file in Locke et al. (2019)

Map shapefile available here: https://doi.org/10.7910/DVN/JNNK7B (Harvard Dataverse)
More information, and national maps, here: https://naturebeyond2020.com/#three_conditions
Map viewable on Google Earth here: http://three-global-conditions.appspot.com/

III. Analysis of ancillary variables across the Three Global Conditions

III.1. List of ancillary variables analyzed

- **land_km2** = Total land area of each DGG cell in km^2, based on Landscan 2007 (Oak Ridge National 2008).
- **popt_2015** = Total 2015 population in persons per DGG cell, from HYDE 3.2.1 (Klein Goldewijk et al. 2017).
- **food_cal** = Total food calories produced (in kcal) within each DGG Cell (Cassidy et al. 2013)
- **pa_km2** = Total protected area within each DGG cell, in km^2, combining the World Database of Protected Areas (WDPA) (UNEP-WCMC 2019) with China protected areas (Li and Shen 2019). Note: may include coastal/marine protected area within DGG cells.
- **kba_km2** = Total Key Biodiversity Area (KBA) in km^2 in each DGG cell (BirdLife International and Conservation International 2018). Note: may include coastal/marine protected area within DGG cells.
- **ind_cnt** = Total Indigenous land area in km^2 within each DGG cell (Garnett et al. 2018).
- **v_rich** = Average number of vertebrate species per 100 km^2 across DGG cell (Jenkins et al. 2013).
- **v_thr** = Average number of threatened vertebrate species per 100 km^2 across DGG cell (Jenkins et al. 2013).
- **FAGBC** = Median forest aboveground biomass carbon density across DGG cell, in Mg/ha (t/ha) (Avitabile et al. 2014).
- **soilC** = Median soil organic carbon density across DGG cell, in Mg/ha (t/ha) (FAO and ITPS 2018).

III. 2. Computation of ancillary variables in DGG cells, using ArcGIS 10.6

1. Compute Total land area within each DGG cell = **land_km2**.
    a. Acquire LANDSCAN 2007 dataset; 30 arc second global raster dataset in geographic format (Oak Ridge National 2008).
    b. Convert DGG shapefile to same raster format as LANDSCAN 2007.
    c. Use zonal stats to compute the total number of 30 arc second cells, and total number of 30 arc second land cells within each DGG cell.
    d. Compute % land area in each DGG cell = land cells / total cells within each DGG cell.
    e. Compute land_km2 = % land area * total land area within each DGG cell in km^2.
2. Compute Total 2015 population within each DGG cell = **popt_2015**.
    a. Acquire 2015 population density per 5M cell, from HYDE 3.2.1 (Klein Goldewijk et al. 2017).
    b. Compute mean population density within each DGG cell using zonal statistics, after converting DGG shapefile into 30 arc second GRID aligned with HYDE GRID.
    c. Compute popt_2015 = population density * land_km2
3. Compute total food calories produced within each DGG cell = **food_cal**.
    a. Acquire data for food calories in 5 arc minute (5M) geographic GeoTiff format (Cassidy et al. 2013), from Earthstat: http://www.earthstat.org/crop-allocation-food-feed-nonfood/.

        i. DeliveredkcalFraction.tif: Fraction of produced calories that are ultimately delivered as food calories after accounting for the type of crop and its allocation as food, feed, or nonfood.
        ii. Glbkcal.tif: Total kilocalories produced for all types of allocations (sum of Food/Feed/NonFood).
    b. Compute GRID for total food calories produced = food_calories = DeliveredkcalFraction * Glbkcal.
    c. Convert to 30s GRID.
    d. Compute mean total food calories for each DGG cell using zonal statistics, after converting DGG shapefile to 30s GRID aligned with total food calories GRID.
    e. Correct for conversion from 5M GRID to DGG format.
        i. Compute correction factor for each DGG cell = 5M area in km^2 / 96 km^2.
        ii. Multiply 5M mean total food calories by the correction factor.
        iii. Product = food_cal. Note that final global computation = 5,677,257,000,000,000 is equivalent to 101.9% of the original global estimate from the dataset and as published.
4. Compute Total protected area within each DGG cell = **pa_km2**.
    a. Acquire WDPA features geodatabase by download from protected planet March 5, 2019. (UNEP-WCMC 2019).
    b. Add to Geodatabase, Intersect WDPA with DGG shapefile, Dissolve to DGG cells.
    c. Acquire dataset for China's protected areas from Xiali Shen (Li and Shen 2019). Shapefile China Reserve.shp.,
    d. Add to Geodatabase, Project to Geographic, Repair geometry.
    e. Intersect China Reserve with DGG shapefile, Dissolve to DGG cells.
    f. Merge with WDPA DGG dataset, Repair features (Production Repair; lots of self-overlaps), Dissolve to DGG cells, Reproject to Eckert IV.
    g. Compute area = pa_km2 = km^2 protected area (WDPA + China) in each DGG cell. Note that these areas may be in error because some DGG cells include coastal marine area, so protected areas may be larger than the land area within DGG cells.
5. Compute Total key biodiversity area (KBA) within each DGG cell = **kba_km2**.
    a. Acquire KBA polygon shapefile by permitted download (May 2019) (BirdLife International and Conservation International 2018).
    b. Add to Geodatabase, Convert Features to Polygons, Repair Geometry.
    c. Intersect with DGG shapefile, Reproject to Eckert IV, Dissolve to DGG cells.
    d. Compute areas = kba_km2 = km^2 = KBA area within DGG cells. Note that these areas may be in error because some DGG cells include coastal marine area, so protected areas may be larger than the land area within DGG cells.
6. Compute Total indigenous land area within each DGG cell = **ind_cnt.**
    a. Obtain dataset as shapefile = IPL_2017.shp from (Garnett et al. 2018).
    b. Convert to 1km raster with GRID environment, Mollweide projection.
    c. Compute total number of 1 km^2 grid cells within each DGG cell using zonal statistics (count), after converting DGG shapefile 1 km raster aligned with Indigenous area GRID.
    d. **ind_cnt** = km^2 indigenous land cells in each DGG cell.
7. Compute Average vertebrate species richness = **v_rich** and threatened vertebrate species = **v_thr**.
    a. Acquire vertebrate data as 10 km resolution raster in Eckert IV GeoTiff format from (Jenkins et al. 2013).
        i. Sum layers for birds, mammals, amphibians.
        ii. vert_rich = number vertebrate species per 10 km cell.
        iii. vert_thr_rich = number threatened vertebrate species per 10 km cell.

b. Convert to Eckert IV 1 km resolution GRID.
c. Convert DGG shapefile to Eckert IV 1 km resolution GRID.
d. Compute mean vert_rich and mean vert_thr_rich within each raster DGG cell using zonal statistics.
    i. v_rich = average number of vertebrate species per 100 km^2 (vert_rich) across each DGG cell.
    ii. v_thr = average number of threatened vertebrate species per 100 km^2 (vert_rich) across each DGG cell.
    iii. Note limited data coverage: >400,000 DGG cells had no data; treated as 0 species.

8. Compute median carbon density across each DGG cell: aboveground forest biomass carbon = **FAGBC** and soil organic carbon = **soilC**.
    a. Acquire datasets
        i. Forest Aboveground Biomass Carbon = GEOCARBON_AGB_Map.tif (Avitabile et al. 2014). 0.01 degree grid cells.
        ii. Soil organic carbon = GSOCmapV1.2.0.tif (FAO and ITPS 2018). 0.0083333333 degree grid cells (30 arc seconds).
    b. Convert floating point rasters to integer rasters to allow median computations.
    c. Create DGG shapefile to rasters with same grid cell format as input datasets (convert features to raster with environment set to input dataset environment.
    d. Compute DGG cell medians using Zotnal stats.
        i. FAGBC = median forest aboveground biomass carbon density across DGG cell, in Mg/ha (t/ha).
        ii. soilC = median soil organic carbon density across DGG cell, in Mg/ha (t/ha).

<u>III. 3. Computation of ancillary variables across the three global conditions</u>

1. Convert all variables for each DGG cell in the DGG shapefile to SPSS format for analysis in SPSS v25, by importing the ".dbf" file of the shapefile into SPSS.
2. Compute statistics for variables that sum to a global total: **land_km2**, **popt_2015, food_cal, pa_km2**, **kba_km2, ind_cnt**
    a. Sum the value of each variable for each DGG cell to compute the total global amount and amount within each condition.
3. Compute statistics for variables that do not sum to a global total: **v_rich, v_thr, FAGBC, soilC.**
    a. Compute area-weighted statistics to correct for variations in land area across each grid cells, producing more reliable statistics for species and carbon densities per unit area.
        i. Select Case weights = pct_land (= land_km2 / DGG cell area in km^2)
        ii. Compute area-weighted statistics.
    b. Use 5% trimmed mean to describe the most common relative values of each variable, to avoid issues with missing data.
    c. Use 5 percentile and 95 percentile to illustrate variable range.

IV. References Cited

Avitabile, V., M. Herold, S. L. Lewis, O. L. Phillips, N. Aguilar-Amuchastegui, G. P. Asner, R. J. W. Brienen, B. R. DeVries, R. G. Gatti, T. R. Feldpausch, C. Girardin, B. de Jong, E. Kearsley, E. Klop, X. Lin, J. Lindsell, G. Lopez-Gonzalez, R. Lucas, Y. Malhi, A. Morel, E. Mitchard, D. Pandey, S. Piao, C. Ryan, M. Sales, M. Santoro, G. Vaglio Laurin, R. Valentini, H. Verbeeck, A. Wijaya, and S. Willcock. 2014. Comparative analysis and fusion for improved global biomass mapping. Pages 251-252 *in* International Conference Global Vegetation Monitoring and Modeling (GV2M), Avignon, France.

BirdLife International, and Conservation International. 2018. Key Biodiversity Area (KBA) digital boundaries: September 2018 version. *in* M. b. B. I. o. b. o. B. I. a. C. International, editor., Downloaded under licence from the Integrated Biodiversity Assessment Tool. http://www.ibatforbusiness.org.

Cassidy, E. S., P. C. West, J. S. Gerber, and J. A. Foley. 2013. Redefining agricultural yields: from tonnes to people nourished per hectare. Environmental Research Letters **8**:034015.

FAO and ITPS. 2018. Global Soil Organic Carbon Map (GSOCmap). FAO, Rome.

Garnett, S. T., N. D. Burgess, J. E. Fa, Á. Fernández-Llamazares, Z. Molnár, C. J. Robinson, J. E. M. Watson, K. K. Zander, B. Austin, E. S. Brondizio, N. F. Collier, T. Duncan, E. Ellis, H. Geyle, M. V. Jackson, H. Jonas, P. Malmer, B. McGowan, A. Sivongxay, and I. Leiper. 2018. A spatial overview of the global importance of Indigenous lands for conservation. Nature Sustainability **1**:369-374.

Jenkins, C. N., S. L. Pimm, and L. N. Joppa. 2013. Global patterns of terrestrial vertebrate diversity and conservation. Proceedings of the National Academy of Sciences **110**:E2602-E2610.

Klein Goldewijk, K., A. Beusen, J. Doelman, and E. Stehfest. 2017. Anthropogenic land use estimates for the Holocene – HYDE 3.2. Earth System Science Data **9**:927-953.

Li, S., and X. Shen. 2019. China protected area dataset comprising boundaries of 456 out of 474 national nature reserves and 752 reserves at provincial and county levels *in*.

Oak Ridge National, L. 2008. LandScan Global Population Database (2007 release, available at: http://www.ornl.gov/sci/landscan/).Oak Ridge National Laboratory, Oak Ridge, TN. Available at http://www.ornl.gov/sci/landscan/

Sahr, K., D. White, and A. J. Kimerling. 2003. Geodesic discrete global grid systems. Cartography and Geographic Information Science **30**:121(114).

Schmill, M. D., L. M. Gordon, N. R. Magliocca, E. C. Ellis, and T. Oates. 2014. GLOBE: Analytics for Assessing Global Representativeness. Pages 25-32 *in* Computing for Geospatial Research and Application (COM.Geo), 2014 Fifth International Conference on.

UN Statistics Division. 2018. Standard country or area codes for statistical use (M49).UN Statistics Division,. Available at https://unstats.un.org/unsd/methodology/m49/overview/

UNEP-WCMC. 2019. The World Database on Protected Areas (WDPA). *in* UNEP-WCMC, editor., Cambridge, UK.

Venter, O., E. W. Sanderson, A. Magrach, J. R. Allan, J. Beher, K. R. Jones, H. P. Possingham, W. F. Laurance, P. Wood, B. M. Fekete, M. A. Levy, and J. E. M. Watson. 2016. Sixteen years of change in the global terrestrial human footprint and implications for biodiversity conservation. Nat Commun **7**:12558.
